# The Virtual Brain: a simulator of primate brain network dynamics

**DOI:** 10.3389/fninf.2013.00010

**Published:** 2013-06-11

**Authors:** Paula Sanz Leon, Stuart A. Knock, M. Marmaduke Woodman, Lia Domide, Jochen Mersmann, Anthony R. McIntosh, Viktor Jirsa

**Affiliations:** ^1^Institut de Neurosciences des Systèmes, Aix Marseille UniversitéMarseille, France; ^2^Department of Neurology, BrainModes Group, Charité University of MedicineBerlin, Germany; ^3^CodemartCluj-Napoca, Romania; ^4^CodeBox GmbHStuttgart, Germany; ^5^Rotman Research Institute at BaycrestToronto, ON, Canada

**Keywords:** connectome, neural masses, time delays, full-brain network model, virtual brain, large-scale simulation, web platform, python

## Abstract

We present The Virtual Brain (TVB), a neuroinformatics platform for full brain network simulations using biologically realistic connectivity. This simulation environment enables the model-based inference of neurophysiological mechanisms across different brain scales that underlie the generation of macroscopic neuroimaging signals including functional MRI (fMRI), EEG and MEG. Researchers from different backgrounds can benefit from an integrative software platform including a supporting framework for data management (generation, organization, storage, integration and sharing) and a simulation core written in Python. TVB allows the reproduction and evaluation of personalized configurations of the brain by using individual subject data. This personalization facilitates an exploration of the consequences of pathological changes in the system, permitting to investigate potential ways to counteract such unfavorable processes. The architecture of TVB supports interaction with MATLAB packages, for example, the well known Brain Connectivity Toolbox. TVB can be used in a client-server configuration, such that it can be remotely accessed through the Internet thanks to its web-based HTML5, JS, and WebGL graphical user interface. TVB is also accessible as a standalone cross-platform Python library and application, and users can interact with the scientific core through the scripting interface IDLE, enabling easy modeling, development and debugging of the scientific kernel. This second interface makes TVB extensible by combining it with other libraries and modules developed by the Python scientific community. In this article, we describe the theoretical background and foundations that led to the development of TVB, the architecture and features of its major software components as well as potential neuroscience applications.

## 1. Introduction

Brain function is thought to emerge from the interaction of large numbers of neurons, under the spatial and temporal constraints of brain structure and cognitive demands. Contemporary network simulations mainly focus on the microscopic and mesoscopic level (neural networks and neural masses representing a particular cortical region), adding detailed biophysical information at these levels of description while too often losing perspective on the global dynamics of the brain. On the other hand, the degree of assessment of global cortical dynamics, across imaging modalities, in human patients and research subjects has increased significantly in the last few decades. In particular, cognitive and clinical neuroscience employs imaging methods of macroscopic brain activity such as intracerebral measurements, stereotactic Encephalography (sEEG) (von Ellenrieder et al., 2012), Electroencephalography (EEG) (Nunez and Srinivasan, 1981; Nunez, 1995; Niedermeyer and Lopes Da Silva, 2005), Magnetoencephalography (MEG) (Hämäläinen, 1992; Hämäläinen et al., 1993; Mosher et al., 1999), and functional Magnetic Resonance Imaging (fMRI) (Ogawa et al., 1993, 1998; Logothetis et al., 2001) to assess brain dynamics and evaluate diagnostic and therapeutic strategies. Hence, there is a strong motivation to develop an efficient, flexible, neuroinformatics platform on this macroscopic level of brain organization for reproducing and probing the broad repertoire of brain dynamics, enabling quick data analysis and visualization of the results.

The Virtual Brain (TVB) is our response to this need. On the one hand, it provides a general infrastructure to support multiple users handling various kinds of empirical and simulated data, as well as tools for visualizing and analyzing that data, either via internal mechanisms or by interacting with other computational systems such as MATLAB. At the same time it provides a simulation toolkit to support a top–down modeling approach to whole brain dynamics, where the underlying network is defined by its structural large-scale connectivity and mesoscopic models that govern the nodes' intrinsic dynamics. The interaction with the dynamics of all other network nodes happens through the connectivity matrix via specific connection weights and time delays, where the latter make a significant contribution to the biological realism of the temporal structure of dynamics.

Historically, Jirsa et al. (2002) first demonstrated neural field modeling on a spherical brain hemisphere employing EEG and MEG forward solutions to obtain simulation imaging signals. In this work, homogeneous (translationally invariant) connectivity was implemented along the lines of Jirsa and Haken (1996, 1997); Bojak and Liley (2010) yielding a neural field equation, which has its roots in classic works (Wilson and Cowan, 1972, 1973; Nunez, 1974; Amari, 1975, 1977). At that time more detailed large-scale connectivity of the full primate brain was unavailable, hence the homogeneous connectivity scaled up to the full brain was chosen as a first approximation (Nunez, 1974). The approach proved successful for the study of certain phenomena as observed in large-scale brain systems including spontaneous activity (Wright and Liley, 1995; Robinson et al., 2001, 2003; Breakspear et al., 2003; Rowe et al., 2004; Freyer et al., 2011), evoked potentials (Rennie et al., 1999, 2002), anesthesia (Liley and Bojak, 2005), epilepsy (Breakspear et al., 2006), sensorimotor coordination (Jirsa and Haken, 1996, 1997), and more recently, plasticity (Robinson, 2011) [see Deco et al. (2008) and Jirsa (2004) for a review].

Careful review of this literature though shows that these models mostly emphasize the temporal domain of brain organization, but leave the spatiotemporal organization untouched. This may be understood by the fact that the symmetry of the connectivity imposes constraints upon the range of the observable dynamics. This was pointed out early by Jirsa et al. (2002) and a suggestion was made to integrate biologically realistic DTI based connectivity into full brain modeling efforts. Large scale brain dynamics are basically expected to reflect the underlying anatomical connectivity between brain areas (Bullmore and Sporns, 2009; Deco et al., 2011), even though structural connectivity is not the only constraint, but the transmission delays play an essential role in shaping the brain network dynamics also (Jirsa and Kelso, 2000; Ghosh et al., 2008; Knock et al., 2009; Jirsa et al., 2010). Recent studies (Pinotsis et al., 2012) have systematically investigated the degree to which homogeneous approximations may serve to understand realistic connection topologies and have concluded that homogeneous approximations are more appropriate for mesoscopic descriptions of brain activity, but less well suited to address full brain network dynamics. All this underscores the need to incorporate realistic connectivity into large scale brain network models. Thus the simulation side of TVB has evolved out of a research program seeking to identify and reproduce realistic whole brain network dynamics, on the basis of empirical connectivity and neural field models (Jirsa and Stefanescu, 2010; Deco et al., 2011).

### 1.1. Modeling

In line with these previous studies, TVB incorporates a biologically realistic, large-scale connectivity of brain regions in the primate brain. Connectivity is mediated by long-range neural fiber tracts as identified by tractography based methods (Hagmann et al., 2008; Honey et al., 2009; Bastiani et al., 2012), or obtained from CoCoMac neuroinformatics database (Kötter, 2004; Kötter and Wanke, 2005; Bakker et al., 2012). In TVB, the tract-lengths matrix of the demonstration connectivity dataset is symmetric due to the fiber detection techniques used to extract the information being insensitive to directionality. On the other hand, the weights matrix is asymmetric as it makes use of directional information contained in the tracer studies of the CoCoMac database. Such details are specific to the connectivity demonstration dataset included in the distribution packages of TVB. The symmetry (or lack thereof) is neither a modeling constraint nor an imposed restriction on the weights and tract-length matrices. The general implementation for weights and tract lengths are full *nodes* × *nodes* matrices without any symmetry restrictions.

Two types of structural connectivity are distinguished in TVB, that is long- and short-range connectivity, given by the connectivity matrix and the folded cortical surface, respectively. The connectivity matrix defines the connection strengths and time delays via finite signal transmission speed between two regions of the brain. The cortical surface allows a more detailed spatial sampling resulting in a spatially continuous approximation of the neural activity as in neural field modeling (Deco et al., 2008; Coombes, 2010; Bressloff, 2012). When using neural mass models, building the network upon the surface allows for the application of arbitrary local connectivity kernels which represent short-range intra-cortical connections. Additionally, networks themselves can be defined at two distinct spatial scales yielding two types of simulations (or brain network models): surface-based and region-based. In the former case, cortical and sub-cortical areas are shaped more realistically, each vertex of the surface is considered a node and is modeled by a neural population model; several nodes belong to a specific brain region, and the edges of the network have a distance of the order of a few millimeters. The influence of delayed activity coming from other brain regions is added to the model via the long-range connectivity. In the latter case of nodes only per region, the connectome itself is used as a coarser representation of the brain network model. The networks comprise discrete nodes, each of which models the neural population activity of a brain region and the edges represent the long-range connectivity (interregional fibers) on the order of a few centimeters. Consequently, in surface-based simulations both types of connectivity, short- and long-range, coexist whereas in region-based simulations one level of geometry is lost: the short-range connectivity.

Neural field models have been developed over many years for their ability to capture the collective dynamics of relatively large areas of the brain in both analytically and computationally tractable forms (Beurle, 1956; Wilson and Cowan, 1972, 1973; Nunez, 1974; Amari, 1975, 1977; Wright and Liley, 1995; Jirsa and Haken, 1996, 1997; Robinson et al., 1997; Jirsa et al., 2002; Atay and Hutt, 2006; Bojak and Liley, 2010). Effectively neural field equations are tissue level models that describe the spatiotemporal evolution of coarse grained variables such as synaptic voltage or firing rate activity in populations of neurons. Some of these models include explicit spatial terms while others are formulated without an explicit spatial component leaving open the possibility to apply effectively arbitrary local connectivity kernels. The lumped representation of the dynamics of a set of similar neurons via a common variable (e.g., mean firing rate and mean postsynaptic potential) is known as neural mass modeling (Freeman, 1975, 1992; Lopes da Silva et al., 1974). Neural mass models accounting for parameter dispersion in the neuronal parameters include Assisi et al., 2005; Stefanescu and Jirsa, 2008, 2011; Jirsa and Stefanescu, 2010. Networks of neural masses, without an explicit spatial component within the mass but with the possibility to apply local connectivity kernels (e.g., Gaussian or Laplacian functions) between masses, can be used to approximate neural field models. Both neural field and neural mass modeling approaches embody the concept from statistical physics that macroscopic physical systems obey laws that are independent of the details of the microscopic constituents of which they are built (Haken, 1983). These and related ideas have been exploited in neurosciences (Kelso, 1995; Buzsaki, 2006).

In TVB, our main interest lies in using the mesoscopic laws governing the behavior of neural populations and uncovering the laws driving the processes on the macroscopic brain network scale. The biophysical mechanisms available to microscopic single neuron approaches are absorbed in the mean field parameters on the mesoscopic scale and are not available for exploration other than through variation of the mean field parameters themselves. As a consequence, TVB represents a neuroinformatics tool that is designed to aid in the exploration of large-scale network mechanisms of brain functioning [see Ritter et al. (2013) for an example of modeling with TVB].

Furthermore, TVB's approach to multi-modal neuroimaging integration in conjunction with neural field modeling shares common features with the work of Bojak et al. (2010, 2011) and Babajani-Feremi and Soltanian-Zadeh (2010). The crucial difference is that the structure upon which TVB has been designed represents a generalized large-scale “computational neural model” of the whole brain. The components of this large-scale model have been separated as cleanly as possible, and a specific structure has been defined for the individual components. This generic structure is intended to serve the purpose of restricting the form of models enough to make direct comparison straight forward while still permitting a sufficiently large class of models to be expressed. Likewise, the paradigms presented during the last few years in this line of research (Sotero et al., 2007; Sotero and Trujillo-Barreto, 2008) could potentially be reproduced, tested and compared in TVB. The mathematics underlying our model-based approach have been partially described in various original articles (Deco et al., 2011; Deco and Jirsa, 2012) and will be reviewed in more detail in future articles.

### 1.2. Informatics

From an informatics perspective, a large-scale simulation project requires a well defined yet flexible workflow, i.e., adaptable according to the users profiles. A typical workflow in TVB involves managing project information, uploading data, setting up simulation parameters (model, integration scheme, output modality), launching simulations (in parallel if needed), analyzing and visualizing, and finally storing results and sharing output data.

The web interface allows users without programming knowledge to access TVB to perform customized simulations (e.g., clinicians could use their patient's data obtained from DTI studies). In addition, it enables them to gain a deeper understanding of the theoretical approaches behind the scenes. On the other hand, theoreticians can design their own models and get an idea of their biophysical realism, their potential physiological applications and implications. As both kinds of users may work within the same framework, the interplay of theory and experiment or application is accelerated. Additionally, users with stronger programming skills benefit from all the advantages provided by the Python programming language: easy-to-learn, easy-to-use, scriptable and with a large choice of scientific modules (Oliphant, 2006).

TVB has been principally built in the Python programming language due to its unique combination of flexibility, existing libraries and the ease with which code can be written, documented, and maintained by non-programmers. The simulation core, originally developed in MATLAB, was ported to Python given its current significance in the numerical computing and neuroscience community and its already proven efficiency for implementing modeling tools (Spacek et al., 2008).

Simulations benefit from vectorized numerical computations with NumPy arrays and are enhanced by the use of the *numexpr* package. Although this allows rather efficient single simulations, the desire to systematically explore the parameter spaces of the neural dynamic models, and to compare many connectivity matrices, has lead to the implementation of code generation mechanisms for the majority of the simulator core—producing C code for both native CPU and also graphics processing units (GPU), leading to a significant speed up of parameter sweeps and parallel simulations (5x from Python to C, 40x from C to GPU). Such graphics units have become popular in scientific computing for their relatively low price and high computing power. Going forward, the GPU implementation of TVB will require testing and optimization before placing it in the hands of users.

This article intends to give a comprehensive but non-exhaustive description of TVB, from both technical and scientific points of view. It will describe the framework's architecture, the simulation core, and the user interfaces. It will also provide two examples, using specific features of the simulator, extracted from the demo scripts which are currently available in TVB's distribution packages.

## 2. TVB architecture

**Figure 1:**
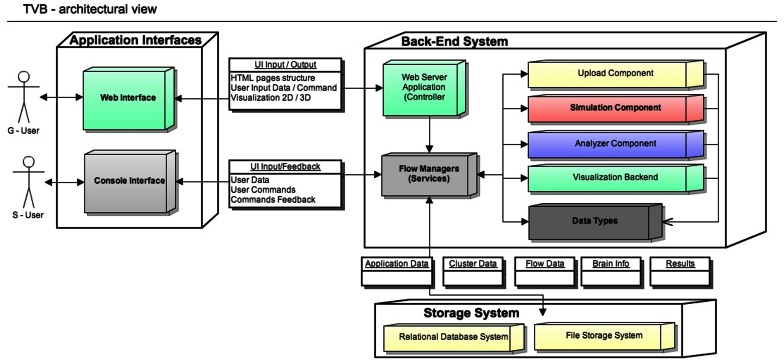
***The Virtual Brain* Architecture: TVB provides two independent interfaces depending on the interaction with users**. Blocks in the back-end are transparently used by different top application layers. TVB-***Datatypes***, are the common language between different components (analyzers, visualizers, simulator, uploaders). They represent “active data” in the sense that, when TVB is configured with a database, data contained in TVB-***Datatypes*** instances are automatically persistent. Currently the console interface works without the storage layer, keeping the results just in memory. S-Users need to manually handle data import and export operations.

The architectural model of the system has two main components: the scientific computing core and the supporting framework with its graphical user interface. Both software components communicate through an interface represented by TVB-***Datatypes***, which are described in section 2.2. In Figure 1 TVB's architectural details are illustrated and explained in more depth.

***General aspects***: TVB is designed for three main deployment configurations, according to the available hardware resources: (1) Stand Alone; (2) Client-Server or, (3) Cluster. In the first, a local workstation is assumed to have certain display, computing power and storage capacity resources. In the second, an instance of TVB is running on a server connected through a network link to client units, and thus accessible to a certain number of users. In this deployment model, simulations use the back-end server's computing power while visualization tasks use resources from the client machine. The third is similar to the client-server configuration, but with the additional advantage of parallelization support in the back-end. The cluster itself needs to be configured separately of TVB.

Based on the usage scenarios and user's level of programming knowledge, two user profiles are represented: a graphical user (G-user) and scripting user (S-user). We therefore provide the corresponding main interfaces based on this classification: a graphical user interface (web) and a scripting interface (IDLE). S-users and G-users have different levels of control over different parts of the system. The profile of S-users is thought to be that of scientific developers, that is, researchers who can elaborate complex modeling scenarios, add their own models or directly modify the source code to extend the scientific core of TVB, mostly working with the scientific modules. They do, nevertheless, have the possibility to enable the database system. In contrast, G-users are relatively more constrained to the features available in the stable releases of TVB, since their profile corresponds more to that of researchers without a strong background in computational modeling. The distinction between these two profiles is mainly a categorization due to the design architecture of TVB. For instance, we could also think of other type of users who want to work with TVB's GUI and are comfortable with programming, and therefore they could potentially make modifications in the code and then see the effect of those when launching the application in a web browser.

The development of TVB is managed under Agile techniques. In accord therewith, each task is considered as *done*, after completing a validation procedure that includes: adding a corresponding automated unit-test, labeling the task as *finished* from the team member assigned to implement the task and further tagging as *closed* from a team member responsible for the module, which means a second level of testing. Before releasing stable packages, there is a period for manual testing, that is, a small group of selected users from different institutions check the main features and functionalities through both interfaces. The navigation and workflows scenarios through the web-based interface are evaluated by means of automated integration tests for web-applications running with Selenium (http://docs.seleniumhq.org/) and Apache-JMeter (http://jmeter.apache.org/) on top of a browser engine. Special effort is being made to provide good code-coverage, including regression tests. Accordingly, the simulation engine of TVB has automated unit-tests, to guarantee the proper and coordinated functioning of all the modules, and simple programs (demonstration scripts), that permit qualitative evaluation of the scientific correctness of results.

The development version of TVB is currently hosted on a private cluster, where we use the version control system *svn* (subversion). Additionally, as any large collaborative open-source project, it is also available in a public repository, using the distributed version control system *git* (Chacon, 2009) to make accessible the scientific core and to gather, manage and integrate contributions from the community. The distribution packages for TVB come with an extensive documentation, including: a *User Guide*, explaining how to install TVB, set up models and run them; *Tutorials, Use Cases* and *Script Demos*, guiding users to achieve predefined simulation scenarios; and a *Developer Guide* and *API reference*. Table 1 provides the links to: the official TVB website, where distribution packages for Linux and Mac OS (32 and 64 bits) and Windows (32 bits) are available for download; the active users group of TVB hosted in Google Groups, where users can ask questions, report issues and suggest improvements or new features; and the public repository, where the source code of both the framework and scientific library (which contains the simulation engine) are available.

**Table 1:** **TVB links**.

TVB official website	http://www.thevirtualbrain.org
Distribution packages	http://www.thevirtualbrain.org/register
Public repository	https://github.com/the-virtual-brain
User group	https://groups.google.com/group/tvb-users/

***Installation and System Requirements***: When using the web interface, users are recommended to have a high definition monitor (at least 1600 × 1000 pixels), a WebGL and WebSockets compatible browser (latest versions of Mozilla Firefox, Apple Safari or Google Chrome), and a WebGL-compatible graphics card, that supports OpenGL version 2.0 or higher (Shreiner et al., 2005).

Regarding memory and storage capacity, for a stand alone installation a minimum of 8 GB of RAM is recommended. For multi-users environments 5 GB of space per user is suggested. This is a storage quota specified by an administrator to manage the maximum hard disk space per user. As for computing power one CPU core is needed for a simulation with a small number of nodes, while simulations with a large number of nodes, such as surface simulations, can make use of a few cores if they are available. When the number of launched simulations is larger than the number of available cores, a serialization is recommended (a serialization mechanism is provided by the supporting framework through the web user interface by specifying the maximum of simultaneous jobs allowed). In order to use the Brain Connectivity Toolbox (Rubinov and Sporns, 2010), MATLAB or Octave should be installed, activated and accessible for the current user.

### 2.1. TVB framework

The supporting framework provides a database back-end, workflow management and a number of features to support collaborative work. The latter feature permits TVB to be setup as a multi-user application. In this configuration, a login system enables users to access their personal sessions; by default their projects and data are private, but they can be shared with other users. The graphical user interface (GUI) is web based, making use of HTML 5, WebGL, CSS3 and Java Script (Bostock et al., 2011) tools to provide an intuitive and responsive interface that can be locally and remotely accessed.

#### 2.1.1. Web-based GUI

**Figure 2:**
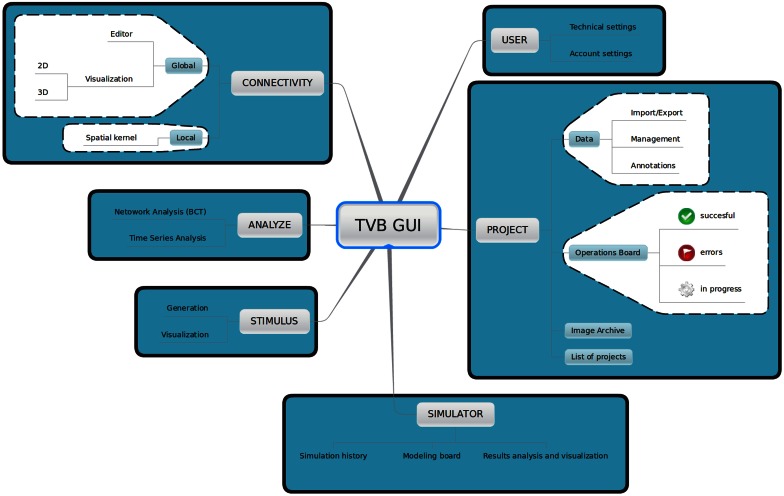
**Main working areas of *The Virtual Brain* 's web interface: in **USER** personal information (account settings) as well as hardware and software preferences (technical settings) are configured**. Through the **PROJECT** area users access and organize their projects, data, figures and the operations dashboard. Input and output simulated data can be exported in HDF5 format and may be used outside of the framework. Brain network models and execution of simulations are configured and launched, respectively in **SIMULATOR**. In this area results can be immediately analyzed and visualized to have a quick overview of the current model. A history of launched simulations is kept to have the traceability of any modifications that took place in the simulation chain. **STIMULUS** provides a collection of tools to build stimulation patterns that will be available to use in the simulations. Finally, **CONNECTIVITY** provides an interactive environment to the edit and visualize connectivity matrices.

**Figure 3:**
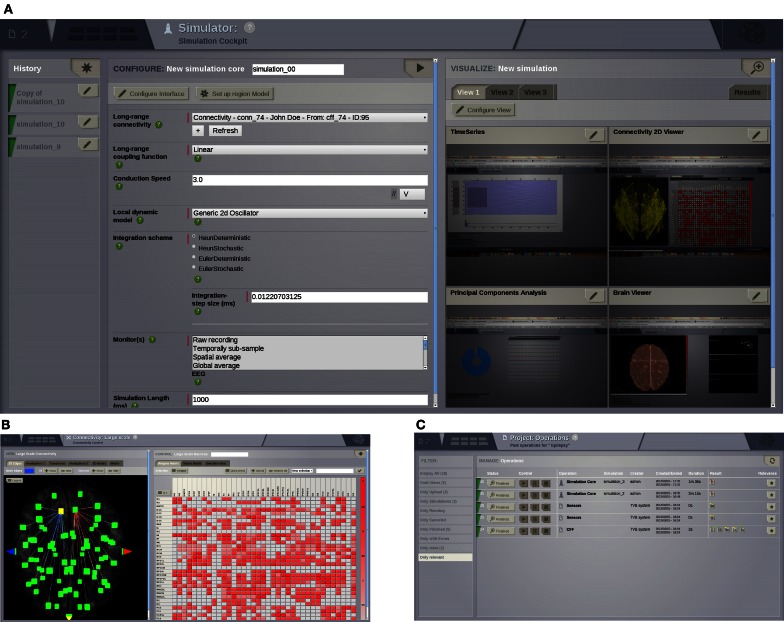
**UI screenshots. (A) SIMULATOR** Area. Having multiple panels allows a quick overview of previous simulations (left), model parameters for the currently selected simulation (middle), and summary displays of the data associated with the currently selected simulation (right). **(B)** Shows the interface for editing and visualising the structural connectivity, for one of the six possible connectivity visualisations. **(C) PROJECT** Area—operations dashboard. On the left column, users can compose filters to search through all the operations on the list.

TVB provides a web-based interactive framework to generate, manipulate and visualize connectivity and network dynamics. Additionally, TVB comprises a set of classic time-series analysis tools, structural and functional connectivity analysis tools, as well as parameter exploration facilities which can launch simulations in parallel on a cluster or on multiple compute cores of a server. The GUI of TVB has six main working areas: **USER, PROJECT, SIMULATOR, ANALYZE, STIMULUS**, and **CONNECTIVITY**. In **USER**, the users manage their accounts and TVB settings. In **PROJECT**, individual projects are managed and navigation tools are provided to explore their structure as well as the data associated with them. A sub-menu within this area provides a dashboard with a list of all the operations along with their current status (running, error, finished), owner, wall-time and associated data, among other information. In **SIMULATOR** the large-scale network model is set up and simulations launched, additional viewers for structural and functional data are offered in 2D and 3D, as well as other displays to visualize the results of a simulation. A history of simulations is also available in this area. In **ANALYZE** time-series and network analysis methods are provided. In **STIMULUS**, users can interactively create stimulation patterns. Finally, in **CONNECTIVITY**, users are given a responsive interface to edit the connectivity matrices assisted by interactive visualization tools. Figure 2 depicts the different working areas, as well as a number of their sub-menus, available through the web UI. A selection of screenshots illustrating the interface in a web browser is given in Figure 3.

#### 2.1.2. Data management and exchange

One of the goals of TVB is to allow researchers from different backgrounds and with different programming skills to have quick access to their simulated data. Data from TVB can be exchanged with other instances of TVB (copies installed on different computers) or with other applications in the neuroscientific community, e.g., MATLAB, Octave, The Connectome ToolKit (Gerhard et al., 2011).

***Export***: A project created within TVB can be entirely exported to a .zip file. Besides storing all the data generated within a particular project in binary files, additional XML files are created to provide a structured storage of metadata, especially with regard to the steps taken to set up a simulation, configuration parameters for specific operations, time-stamps and user account information. This mechanism produces a summary of the procedures carried on by researchers within a project which is used for sharing data across instances of TVB. The second export mechanism allows the export of individual data objects. The data format used in TVB is based on the HDF5 format (The HDF Group, 2010) because it presents a number of advantages over other formats: (1) huge pieces of data can be stored in a condensed form; (2) it allows grouping of data in a tree structure; (3) it allows metadata assignment at every level; and (4) it is a widely used format, accessible in several programming languages and applications. Additionally, each object in TVB has a global unique identifier (GUID) which makes it easy to identify an object across systems, avoiding naming conflicts among files containing objects of the same type.

***Import***: A set of mechanisms (“uploaders”) is provided in TVB to import data into the framework, including neuroimaging data generated independently by other applications. The following formats are supported: NIFTI-1 (volumetric time- series), GIFTI (surfaces) and CFF (connectome file). General compression formats, such as ZIP and BZIP2 are also supported for certain data import routines that expect a set of ASCII text files compressed in an archive. Hence the use of general compression formats means that preparing datasets for TVB is as simple as generating an archive with the correct ASCII files, in contrast to some of the other neuroscientific data formats found elsewhere. For instance, a ***Connectivity*** dataset (*connectome*) may be uploaded as a zip folder containing the following collection of files: (1) areas.txt, (2) average_orientations.txt, (3) info.txt, (4) positions.txt, (5) tract_lengths.txt, and (6) weigths.txt. More conventions and guidelines to use each uploader routine can be found in the *User Guide* of TVB's documentation.

#### 2.1.3. File storage

The storage system is a tree of folders and files. The actual location on disk is configurable by the user, but the default is a folder called “TVB” in the user's home folder. There is a sub-folder for each **Project** in which an XML file containing details about the project itself is stored. Then for each operation, one folder per operation is created containing a set of .h5 files generated during that particular operation, and one XML file describing the operation itself. The XML contains tags like *creation date, operation status* (e.g., Finished, Error), *algorithm reference, operation GUID*, and most importantly *input parameters dictionary*. Sufficiently detailed information is stored in the file system to be able to export data from one instance of TVB and to then import it into another instance, correctly recreating projects, including all operations and their results. Even though the amount of data generated per operation varies greatly, since it depends strongly on the Monitors used and parameters of the simulation, some rough estimates are given below:

- A 1000 ms long, region-based simulation with all the default parameters requires approximatively 1 MB of disk space.
- A 10 ms long, surface-based simulation, using a precalculated sparse matrix to describe the local connectivity kernel and all the default parameters, requires about 280 MB.

Users can manually remove unused data using the corresponding controls in TVB's GUI. In this case, all files related to these data are also deleted, freeing disk space. The amount of physical storage space available to TVB can be configured in the **USER → Settings** working area of the GUI—this is, of course, limited by the amount of free space available on the users hard drives.

#### 2.1.4. Database management system

Internally, TVB framework uses a relational database (DB), for ordering and linking entities and as an indexing facility to quickly look up data. At install time, users can choose between SQLite (a file based database and one of the most used embedded DB systems) and PostgreSQL (a powerful, widely spread, open-source object-relational DB system which requires a separate installation by users) as the DB engine. In the database, only references to the entities are stored, with the actual operation results always being stored in files, due to size. A relational database was chosen as it provides speed when filtering entities and navigating entity relationship trees.

### 2.2. TVB datatypes

In the architecture of TVB, a middleware layer represented by TVB-***Datatypes*** allows the handling and flow of data between the scientific kernel and the supporting framework. TVB-***Datatypes*** are annotated data structures which contain one or more data attributes and associated descriptive information, as well as methods for operating on the data they contain. The definition of a ***Datatype*** is achieved using TVB's traiting system, which was inspired by the traiting system developed by Enthought (Enthought, 2001). The traiting system of TVB, among other things, provides a mechanism for annotating data, that is, associating additional information with the data which is itself usually a single number or an array of numbers. A complete description of TVB's traiting system is beyond the scope of this article. However, in describing TVB's ***Datatypes*** we will give an example of its use, which should help to provide a basic understanding of the mechanism.

A number of basic TVB-***Datatypes*** are defined based on Types that are part of the traiting system, with these traited Types, in turn, wrapping Numpy data types. For instance, TVB-***FloatArray*** is a datatype derived from the traiting system's Array type, which in turn wraps Numpy's **ndarray**. The traiting system's Array type has attributes or annotations, such as: dtype, the numerical type of the data contained in the array; label, a short (typically one or two word) description of what the Array refers to, this information is used by the supporting framework to create a proper label for the GUI; doc, a longer description of what the Array refers to, allowing the direct integration of useful documentation into array objects; and default, the default value for an instance of an Array type. In the case of a ***FloatArray***, the dtype attribute is fixed as being numpy.float64.

More complex, higher-level, TVB-***Datatypes*** are then built up with attributes that are themselves basic TVB-***Datatypes***. For example, TVB-***Connectivity*** is datatype which includes multiple ***FloatArray***s, as well as a number of other traited types, such as Integer and Boolean, in its definition. An example of a ***FloatArray*** being used to define an attribute of a ***Connectivity*** can be seen in Code 1. The high-level ***Datatypes*** currently defined in TVB are summarized in Table 2.

Code 1 **An instance of TVB's *FloatArray Datatype* being used to define the conduction speed between brain regions as an attribute of a *Connectivity Datatype*.**

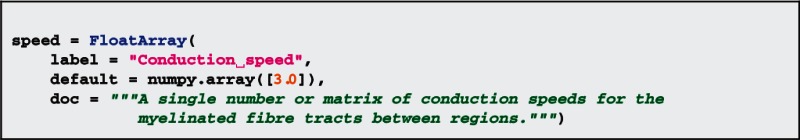

**Table 2:** **TVB Datatypes**.

**Base class datatype**	**Description**	**Derived classes**
Connectivity	Maps connectivity matrix data	Connectivity
Surfaces	Covers surface representations	CorticalSurface, SkinAir, BrainSkull, SkullSkin, EEGCap, FaceSurface, Cortex, RegionMapping, LocalConnectivity
Volumes	Wraps volumetric data	ParcellationMask, StructuralMRI
Sensors	Wraps sensors data used in different acquisition techniques to generate physiological recordings	SensorsEEG, SensorsMEG, SensorsInternal
ProjectionMatrix	Wraps matrices defining a linear operator to map the spatial sources into the leadfield domain	ProjectionRegionEEG, ProjectionSurfaceEEG, ProjectionRegionMEG
	It relates two datatypes: a source of type Connectivity or Surface and a set of Sensors	ProjectionSurfaceMEG
	The matrix is computed using OpenMEEG. (Gramfort et al., 2010)	
Equations	Commonly used functions for defining local connectivity kernels and stimulation patterns	
SpatialPattern	Contains patterns mainly used as stimuli. It makes use of Equation datatypes	SpatioTemporalPattern, StimuliRegion, StimuliSurface, SpatialPatternVolume
TimeSeries	One of the most important TVB-Datatypes. Derived classes wrap measurements recorded under different acquisition modalities	TimeSeriesRegion, TimeSeriesSurface, TimeSeriesVolume, TimeSeriesEEG, TimeSeriesMEG
Graph	Wraps results from a covariance analysis or results from BCT analyzers	Covariance, ConnectivityMeasure
MappedValues	Wraps a single value computed from a TimeSeries object	
ModeDecomposition	Wraps results from matrix factorization analysis (i.e., PCA and ICA)	PrincipalComponents, IndependentComponents
Spectral	Wraps results from frequency analysis	FourierSpectrum, WaveletCoefficients, ComplexCoherenceSpectrum

Specifications about the requirements to build a TVB-Datatype can be found in the documentation of the distribution packages.

An example indicating the usage and features of TVB-***Datatypes*** is provided below. When a user uploads a connectivity dataset through the UI, an instance of a ***Connectivity*** datatype is generated. This ***Connectivity*** datatype is one of the required input arguments when creating an instance of Simulator. As a result of the execution of a simulation, other TVB-***Datatypes*** are generated, for instance one or more ***TimeSeries*** datatypes. Specifically, if the simulation is run using the MEG and EEG recording modalities then *TimeSeriesMEG*, *TimeSeriesEEG*, which are subclasses of ***TimeSeries***, are returned. Both the ***Connectivity*** and ***TimeSeries*** datatypes are accepted by a range of appropriate analysis and visualization methods.

Further, TVB-***Datatypes*** have attributes and metadata which remains accessible after exporting in TVB format. The metadata includes a technical description of the data (storage size for instance) as well as scientifically relevant properties and useful documentation to properly interpret the dataset. In the shell interface, the attributes of TVB-***Datatype*** can be accessed by their key-names in the same way as Python dictionaries.

### 2.3. TVB simulator

**Figure 4:**
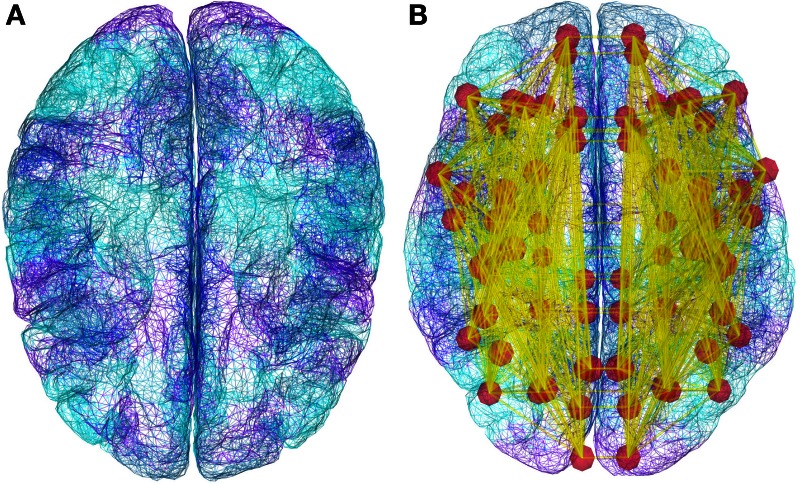
**Demonstration datasets exist in TVB for the anatomical structure on which simulations are built, including a triangular mesh surface representation of the neocortex **(A)** and white matter fiber lengths **(B)****. However, new data from structural imaging such as MRI, DTI, and DSI for individual subjects, as well as data from the literature can be used and wrapped in a TVB-***Datatype***.

The simulation core of TVB brings together a mesoscopic model of neural dynamics with structural data. The latter defines both the spatial support (see Figure 4), upon which the brain network model is built, and the hierarchy of anatomical connectivity, that determines the spatial scale represented by the structural linkages between nodes (Freeman, 1975). Simulations then recreate the emergent brain dynamics by numerically integrating this coupled system of differential equations. All these entities have their equivalent representation as *classes* either in the scientific modules or ***datatypes***, and are bound together in an instance of the *Simulator* class. In the following paragraphs we describe all the individual components required to build a minimal representation of a brain network model and run a simulation, as well as the outline of the operations required to initialize a *Simulator* object and the operations of the update scheme.

#### 2.3.1. Coupling

The brain activity (state variables) that has been propagated over the long-range ***Connectivity*** pass through these functions before entering the equations of a *Model* describing the local dynamics. A *Coupling* function's primary purpose is to rescale the incoming activity to a level appropriate to the population model. The base *Coupling* class as well as a number of different coupling functions are implemented in the coupling module, for instance *Linear* and *Sigmoidal*.

#### 2.3.2. Population models

A set of default mesoscopic neural models are defined in TVB's models. All these models of local dynamics are classes derived from a base *Model* class.

We briefly discuss the implemented population models in order of increasing complexity. They include a generic two dimensional oscillator, a collection of classical population models and two recently developed multi-modal neural mass models. Below, *N* refers to the number of state variables or equations governing the evolution of the model's temporal dynamics; *M* is the number of modes and by default *M* = 1 except for the multi-modal models.

The *Generic2dOscillator* model (*N* = 2) is a generic phase-plane oscillator model capable of generating a wide range of phenomena observed in neuronal population dynamics, such as multistability, the coexistence of oscillatory and non-oscillatory dynamics, as well as displaying dynamics at multiple time scales.

The *WilsonCowan* model (Wilson and Cowan, 1972) (*N* = 2) describes the firing rate of a neural population consisting of two subpopulations (one excitatory and the other inhibitory). It was originally derived using phenomenological arguments. This neural mass model provides an intermediate between a microscopic and macroscopic level of description of neural assemblies and populations of neurons since it can be derived from pulse-coupled neurons (Haken, 2001) and its continuum limit resembles neural field equations (Jirsa and Haken, 1996).

The *WongWang* model (Wong and Wang, 2006) represents a reduced system of *N* = 2 coupled non-linear equations, originally derived for decision making in two-choice tasks. The *BrunelWang* model (Brunel and Wang, 2001, 2003) is a mean field model derived from integrate-and-fire spiking neurons and makes the approximation of randomly distributed interspike intervals. It is notable that this population model shows only attractor states of firing rates. It has been extensively used to study working memory. Its complexity resides in the number of parameters that it uses to characterize each population (*N* = 2). These parameters correspond to physical quantities that can be measured in neurophysiology experiments. The current implementation of this model is based on the approach used in (Deco and Jirsa, 2012).

The *JansenRit* model (Jansen and Rit, 1995) is a derivative of the Wilson-Cowan model and features three coupled subpopulations of cortical neurons: an excitatory population of pyramidal cells interacting with two populations of interneurons, one inhibitory and the excitatory. This model can produce alpha activity consistent with that measured in EEG, and is capable of simulating evoked potentials (Jansen et al., 1993). It displays a surprisingly rich and complex oscillatory dynamics under periodic stimulation (Spiegler et al., 2010). Each population is described by a second order differential equation. As a consequence the system is described by a set of *N* = 6 first order differential equations.

The *StefanescuJirsa2D* and *StefanescuJirsa3D* models (Stefanescu and Jirsa, 2008; Jirsa and Stefanescu, 2010; Stefanescu and Jirsa, 2011) are neural mass models derived from a globally coupled population of neurons of a particular kind. The first one has been derived from coupled FitzHugh-Nagumo neurons (FitzHugh, 1961; Nagumo, 1962), which, with *N* = 2, are capable of displaying excitable dynamics, as well as oscillations. The second is derived from coupled Hindmarsh-Rose neurons (Hindmarsh and Rose, 1984), which are also capable of producing excitable and oscillatory dynamics, but with *N* = 3 have the additional capability of displaying transient oscillations and bursts. The two Stefanescu-Jirsa models show the most complex repertoire of dynamics (including bursting and multi-frequency oscillations). They have been derived using mean field techniques for parameter dispersion (Assisi et al., 2005) and have an additional dimension, the mode *M*, which partitions the dynamics into various subtypes of population behavior. These models are therefore composed of 12 (*N* = 4, *M* = 3) and 18 (*N* = 6, *M* = 3) state variables, respectively.

#### 2.3.3. Integrators

The base class for integration schemes is called *Integrator*, an integrators module contains this base class along with a set of specific integration scheme classes for solving both deterministic and stochastic differential equations. The specific schemes implemented for brain network simulations include the *Euler* and *Heun* methods. The 4th-order *Runge-Kutta* (rk4) method is only available for solving ordinary differential equations (ODEs), i.e., deterministic integration, given that there are various variants for the stochastic version of the method, differing rates of convergence being one of the points that several attempts of creating a stochastic adaptation fail at [see Burrage et al. (2004) for an overview]. Therefore, this method is available for drawing example trajectories in the interactive phase-plane plot tool.

#### 2.3.4. Noise

Noise plays a crucial role for brain dynamics, and hence for brain function (McIntosh et al., 2010). The Noise module consists of two base classes: *RandomStream* that wraps Numpy's RandomState class and *Noise*. The former provides the ability to create multiple random streams which can be independently seeded or set to an explicit initial state. The latter is the base class from which specific noises, such as white and colored (Fox et al., 1988), are derived. In TVB's implementation *Noise* enters as an additional term within the stochastic integration schemes, and can be either an *Additive* or *Multiplicative* process (Klöden and Platen, 1995). As well as providing a means to generate reproducible stochastic processes for the integration schemes, the related classes in noise are used to set the initial conditions of the system when no explicit initial conditions are specified.

#### 2.3.5. Monitors

**Figure 5:**
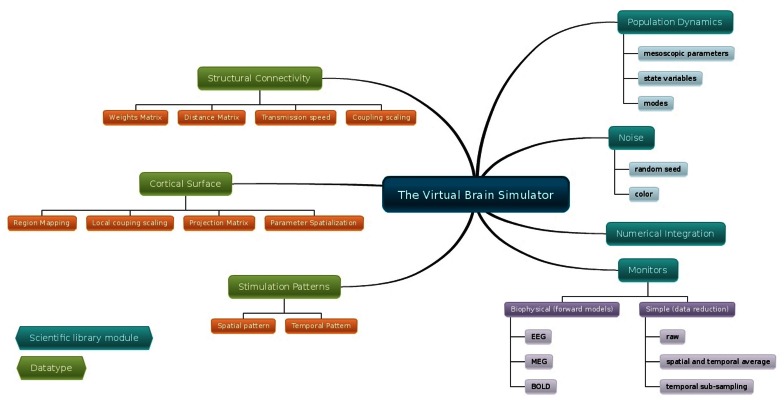
**Diagram of the configurable elements for building a brain network model and launching a simulation**. TVB can incorporate cortical connectivity information from an individual's tractographic and cortical geometry data. The ***Connectivity*** object contains matrices defining the connection strengths and time delays via finite signal transmission speed between all regions, while the folded ***Cortical Surface*** mesh provides the spatial support for finer resolution models. In the latter case a ***Local Coupling*** defines the interaction between neighboring nodes. In its simplest form local connectivity is spatially invariant, however, support exists for spatial inhomogeneity. Signal propagation via local connectivity is instantaneous (no time delays), which is a reasonable approximation considering the short distances involved. Together, the cortical surface with its local connectivity, the long-range connectivity matrix, and the neural mass models defining the local dynamics define a full brain network model. Additionally, stimulation can be applied to a simulation. The stimulation patterns are built in terms of spatial and temporal equations chosen independently. For region-based network models, it is only possible to build time dependent stimuli since there is not a spatial extent for a region node. However, node-specific weightings can be set to modulate the intensity of the stimulus applied to each node. For surface-based models, equations with finite spatial support are evaluated as a function of distance from one or more focal points (vertices of the surface), where the equation defines the spatial profile of the stimuli. The neural source activity from both region or surface-based approaches can be projected into EEG, MEG and BOLD (Buxton and Frank, 1997; Friston et al., 2000) space using a forward model (Breakspear and Jirsa, 2007).

The data from a simulation is processed and recorded while the simulation is running, that is, while the differential equations governing the system are being integrated. The base class for these processing and recording methods is the *Monitor* class in the Monitors module. We consider two main types of online-processing: (1) raw or low-level; and (2) biophysical or high-level. The output of a *Monitor* is a 4-dimensional array (which can be wrapped in the corresponding ***TimeSeries*** datatype), i.e., a 3D state vector as a function of time. For the first kind of *Monitors* these dimensions correspond to [*time, state variables, space, modes*] where “space” can be either brain regions or vertices of a cortical surface plus non-cortical brain regions. The number of state variables as well as the number of modes strictly depend on the *Model*. For the second kind of *Monitors*, the dimensions are [*time*, 1, *sensors*, 1]. The simplest form of low-level *Monitor* returns all the simulated data, i.e., time points are returned at the sampling rate corresponding to the integration scheme's step size and all state variables are returned for all nodes. All other low-level *Monitors* perform some degree of down-sampling, such as returning only a reduced set state variables (by default the *variables of interest* of a *Model*), or down-sampling in “space” or time. Some variations include temporally sub-sampled, spatially averaged and temporally sub-sampled, or temporally averaged. The biophysical *Monitors* instantiate a physically realistic measurement process on the simulation, such as *EEG*, *MEG*, *SEEG* or *BOLD*. For the first two, a ***ProjectionMatrix*** is also required. This matrix maps source activity (“space”) to sensor activity (“sensors”). OpenMEEG (Gramfort et al., 2010) was used to generate the demonstration projection matrix, also known as lead-field or gain matrix, that corresponds to the EEG/MEEG forward solution. The forward solution modeling the signals from depth electrodes is based on the point dipole model in homogeneous space (Sarvas, 1987). The *BOLD* monitor is based on Buxton and Frank (1997) and Friston et al. (2000). Figure 5 summarizes the fundamental blocks required to configure a full model, launch a simulation and retrieve the simulated data.

In most neural mass models there is a state variable representing some type of neural activity (firing rate, average membrane potential, etc.), which serves as a basis for the biophysical monitors. The state variables used as source of neural activity depend both on the *Model* and the biophysical space that it will be projected onto (MEG, EEG, BOLD). Given a neural mass model with a set of state variables, G-Users can choose which subset of state variables will be fed into a *Monitor* (independently for each monitor). However, how a given *Monitor* operates on this subset of state variables is an intrinsic property of the monitor. Users with programming experience can, of course, define new monitors according to their needs. Currently, there is not a mechanism providing automatic support for general operations over state variables before they are passed to a monitor. As such, when the neural activity entering into the monitors is anything other than a summation or average over state variables then it is advised to redefine the *Model* in a way that one of the state variables actually describes the neural activity of interest.

#### 2.3.6. Outline of the simulation algorithm

The *Simulator* class has several methods to set up the spatiotemporal dimensions of the input and output arrays, based on configurable attributes of the individual components such as integration time step (e.g., integrators.*HeunDeterministic*.dt), structural spatial support (e.g., ***connectivity***.*Connectivity* or ***surfaces***. *CorticalSurface*) and transmission speed (e.g., ***connectivity***.*Connectivity*.speed) as well as a cascade of specific configuration methods to interface them. The *Simulator* class coordinates the collection of objects from all the modules in the scientific library needed to build the network model and yield the simulated data. To perform a simulation a *Simulator* object needs to be: (1) configured, initializing all the individual components and calculating attributes based on the combination of objects passed to the *Simulator* instance; and (2) called in a loop to obtain simulated data, i.e., to run the simulation (see Code 2). The next paragraphs list the main operations of the simulation algorithm.

Code 2 **Script example to simulate 1 second of brain activity. Output is recorded with two different monitors.**

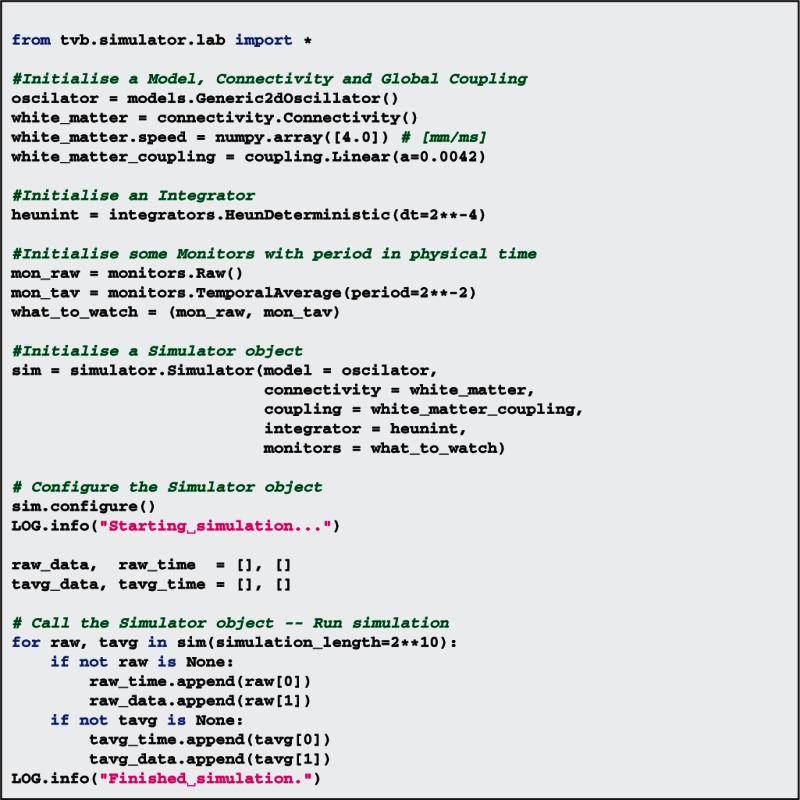

***Initializing a*Simulator**

1. Check if the transmission speed was provided.
2. Configure the ***Connectivity*** matrix (connectome). The delays matrix is computed using the distance matrix and the transmission speed. Get the number of regions.
3. Check if a ***Surface*** is provided.
4. Check if a stimulus ***pattern*** is provided.
5. Configure individual components: *Model*, *Integrator*, *Monitors*. From here we obtain integration time step size, number of statevariables, number of modes.
6. Set the number of nodes (region-based or surface-based simulation). If a ***Surface*** was given the number of nodes will correspond to the number of vertices plus the number of non-cortical regions, otherwise it will be equal to the number of regions in the ***Connectivity*** matrix.
7. Spatialise model parameters if required. Internally, TVB uses arrays for model parameters, if the size of the array for a particular parameter is 1, then the same numerical value is applied to all nodes. If the size of the parameter array is *N*, where *N* is the number of nodes, the parameter value for each node is taken from the corresponding element of the array of parameter values.
8. If applicable, configure spatial component of stimulation ***Patterns*** (requires number of nodes).
9. Compute delays matrix in integration time steps.
10. Compute the horizon of the delayed state, that is the maximum delay in integration time steps.
11. Set the history shape. The history state contains the activity that propagates from the delayed state to the next.
12. Determine if the *Integrator* is deterministic or stochastic. If the latter, then configure the *Noise* and the integration method accordingly.
13. Set initial conditions. This is the state from which the simulation will begin. If none is provided, then random initial conditions are set based on the ranges of the model's state variables. Random initial conditions are fed to the initial history array providing the minimal state of the network with time-delays before *t* = 0. If initial conditions are user-defined but the length along the time dimension is shorter than the required horizon, then the history array will be padded using the same method of described for random initial conditions.
14. Configure the monitors for the simulation. Get variables of interest.

***Calling a*Simulator**

1. Get simulation length.
2. Compute estimates of run-time, memory usage and storage.
3. Check if a particular random state was provided (random seed). This feature is useful for reproducibility of results, for instance, getting the same stream of random numbers for the *Noise*.
4. Compute the number of integration steps.
5. If the simulation is surface-based, then get attributes required to compute ***Local Connectivity*** kernel.
6. Update state loop:
  - Get the corresponding coupled delayed activity. That is, compute the dot product between the weights matrix (connectome) and the delayed state of the coupling variables, transformed by a (long-range) *Coupling* function.
  - Update the state array. This is the numerical integration, i.e., advancing an integration time step, of the differential equations defining the neuron model. Distal delayed activity, local instantaneous activity and stimulation are fed to the integration scheme.
  - Update the history.
  - Push state data onto the *Monitors*. Yield any processed time-series data point if available.

As a working example, in Code 2, we show a code snippet which uses TVB's scripting interface and some of the classes and modules we have just described to generate one second of brain activity. The for loop in the example code allows scripting users to receive time-series data as available and separately for each of the monitors processing simulated raw data. In this implementation, at each time step or certain number of steps, data can be directly stored to disk, reducing the memory footprint of the simulation. Such a feature is particularly useful when dealing with larger simulations. Likewise, data can be accessed while the simulation is still running, which proves to be advantageous for modeling paradigms where one of the output signals is fed back to the network model as stimulation for instance (see the paragraph about *Dynamic modeling* in section 3).

### 2.4. Analyzers and visualizers

For the analysis and visualisation of simulated neuronal dynamics as well as imported data, such as anatomical structure and experimentally recorded time-series, several algorithms and techniques are currently available in TVB. Here we list some of the algorithms and methods that are provided to perform analysis and visualization of data through the GUI.

***Analyzers*** are mostly standard algorithms for time-series and network analysis. The analyzers comprise techniques wrapping functions from Numpy (Fast Fourier Transform (FFT), auto-correlation, variance metrics), Scipy (cross-correlation), scikit-learn (ICA) (Pedregosa et al., 2011) and matplotlib-mlab (PCA) (Hunter, 2007). In addition, there are specific implementations of the wavelet transform, complex coherence (Nolte et al., 2004; Freyer et al., 2012) and multiscale entropy (MSE) (Costa et al., 2002, 2005; Lake and Moorman, 2011).

**Figure 6:**
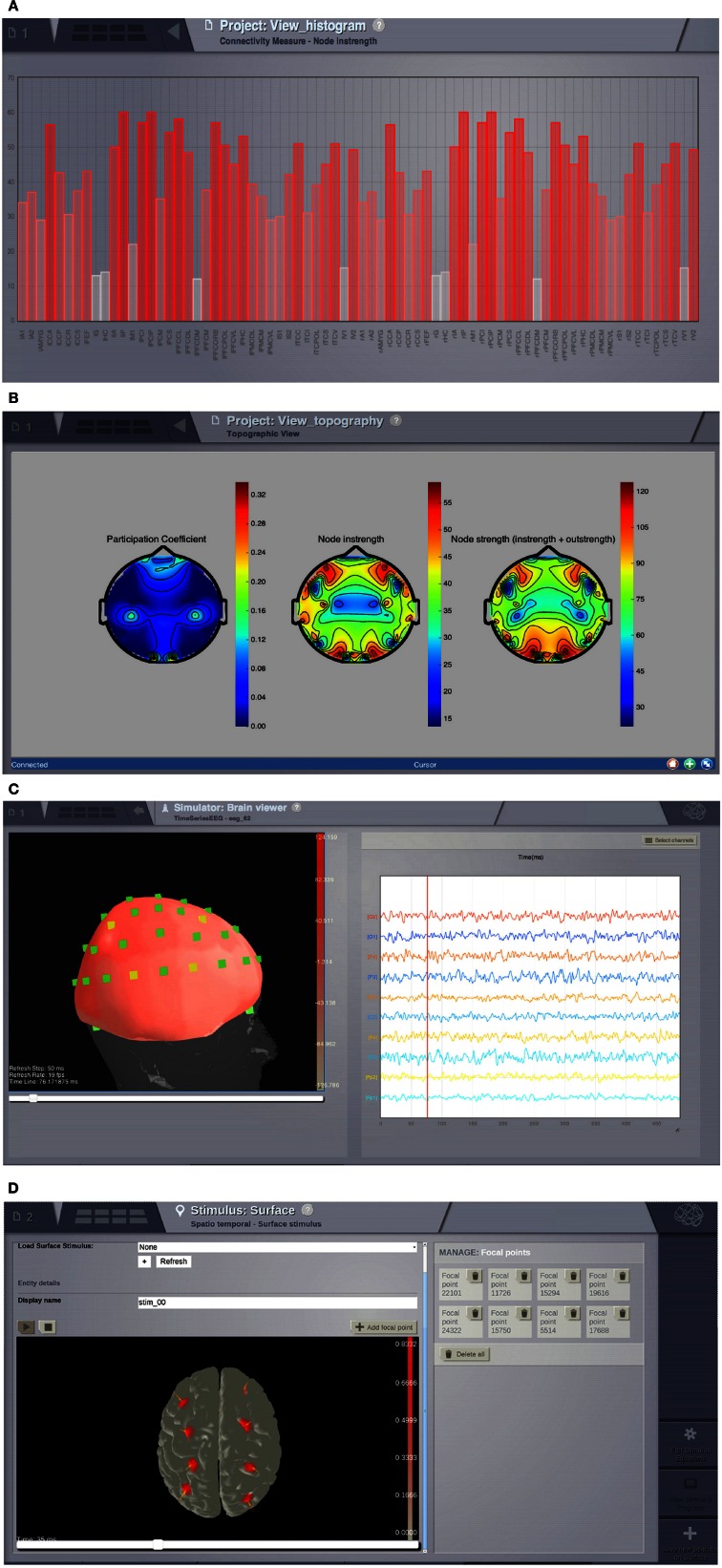
**Visualizers. (A)** Histogram of a graph metric as a function of nodes in the connectivity matrix. **(B)** A 2D projection of the head. The color map represents a graph metric computed on the connectivity matrix. **(C)** EEG visualizer combines a rendered head surface, an overlay with the sensors positions and an interactive time-series display. **(D)** An animated display of the spatiotemporal pattern applied to the cortical surface. Red spots represent the focal points of the spatial component of the stimulus.

***Visualizers*** are tools designed to correctly handle specific ***datatypes*** and display their content. Representations currently available in the GUI include: histogram plots (Figure 6A); interactive time-series plots, EEG (Figure 6C); 2D head topographic maps (Figure 6B); 3D displays of surfaces and animations (Figure 6D) and network plots. Additionally, for shell users there is a collection of plotting tools available based on matplotlib and mayavi (Ramachandran and Varoquaux, 2011).

## 3. Performance, reproducibility, and flexibility

### 3.1. Testing for speed

In the context of full brain models there is no other platform against which we could compare the performance results for TVB and define a good ratio run-time/real-time. As a first approximation a simple network of 74 nodes, whose node dynamics were governed by the equations of the *Generic2dOscillator* model (see Code 3) was implemented in the Brian spiking neural network simulator. The integration step size was 0.125 ms (*dt* = 2^−3^ ms) and the simulation length was 2048 ms. This network was evaluated without time delays and using a random sparse connectivity matrix. Execution times were about 4.5 s in Brian and 15 s in TVB. In contrast, when heterogeneous time delays were included, running times of the simulations implemented in Brian increased considerably (approximately 6.5x) whereas in TVB they hardly changed (approximately 1.2x). Simulations were run on a CPU Intel® Xeon ® W3520 @ 2.67 GHz. These results, although informative, expose the fact that the architectures of TVB and the Brian simulator are different and therefore they have been optimized accordingly to serve distinct purposes from a modeling point of view.

Code 3 **State equations of the generic plane oscillator as scripted to run the simulation in the Brian simulator. The description of the parameters are explained in the API documentation and will be discussed in the context of dynamical systems elsewhere.**

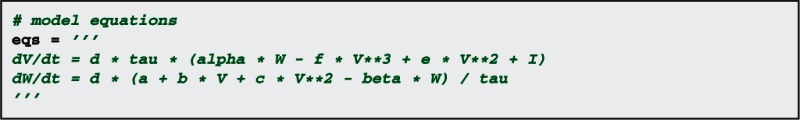

**Figure 7:**
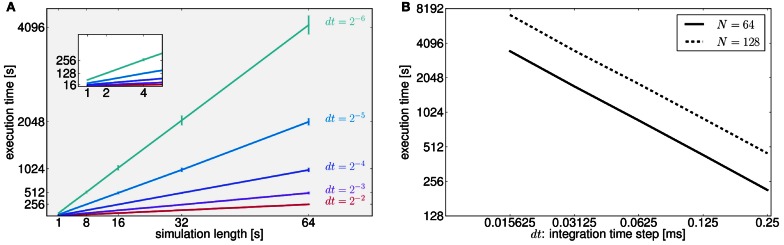
**(A)** As expected for fixed time-step schemes, execution times scale linearly with the number of integration steps. We used seven values of simulation lengths (1, 2, 4, 8, 16, 32, and 64 s) and five values of integration time step (*dt* = 2^−2^ = 0.25, *dt* = 2^−3^ = 0.125, *dt* = 0.0625 = 2^−4^, *dt* = 0.03125 = 2^−5^, and *dt* = 0.015625 = 2^−6^ ms). For each possible combination 100 simulations were performed. The network model consisted of 74 nodes (with two state variables and one mode per node). Numerical integration was based on Heun's stochastic method. We plot the average execution time with the error bars representing the standard deviation over simulations. The inset shows a narrower range for simulation lengths between 1 and 4 s. Axes units and color code are the same as those displayed in the main plot. **(B)** Here, execution times are shown as a function of the integration time step size, *dt*, for two different number of nodes (solid and dashed lines correspond to connectivity matrices of 64 and 128 nodes, respectively) for a specific conduction speed (4 mm/ms) and simulation length (64 s). Both axes are in logarithmic scale with base 2. In this case, halving *dt* or doubling the number of nodes in the connectivity matrix, *N*, doubles the running time. However, as mentioned in the text, for larger networks execution times seem to grow quadratically as a function of the number of nodes in the network. Further tests need to be developed to understand this behavior.

To assess the performance of TVB in terms of simulation timings, we also ran simulations for all possible combinations of two parameters: simulation length and integration time step (Figure 7A). We made the following estimates: it takes on average 16 s to compute 1 s of brain network dynamics [at the region level, with an integration time step of 0.0625 ms (*dt* = 2^−4^ ms) and including time delays of the order of 20 ms which amounts to store about 320 past states per time step] on CPUs Intel ® Xeon ® X5672 @ 3.20 GHz, CPU cache of 12 MB and Linux kernel 3.1.0-1-amd64 as operating system. In Figure 7B we quantify how running times increase as a function of the integration time step in 64 s long (region-based) simulations for two different sizes of the connectivity matrix.

In general, human cortical connectomes are derived from anatomical parcellations with a variable number of nodes, from less than 100 to over a few thousands nodes (Zalesky et al., 2010). Preliminary results of simulations (data not shown) using connectivity matrices of different sizes (16, 32, 64, 128, 256, 512, 1024, 2048, and 4096 nodes) and a supplementary parameter (transmission speed that has an effect on the size of the history array keeping the delayed states of the network) indicate that there is a quadratic growth of the running times for networks with more than 512 nodes. Since performance depends on a large number of parameters which have an effect on both memory (CPU cache and RAM) and CPU usage, and therefore resulting running times arise from the interaction between them, we see the need to develop more tests to stress in particular memory capacity and bandwidth in order to fully understand the aforementioned behavior.

In Future Work we talk about the approaches to benchmark and improve the execution times of simulations. For the present work we have restricted ourselves to present performance results looking at the parameters that have the strongest effect on simulations timings.

### 3.2. Reproducibility of results from the literature

**Figure 8:**
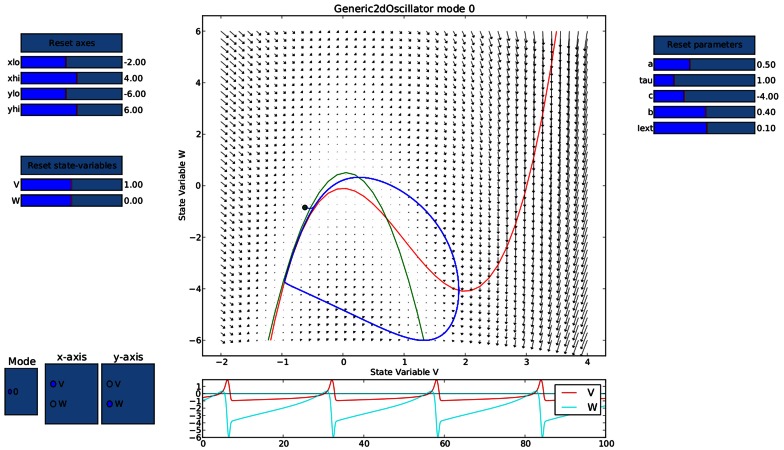
**Phase portrait using TVB's interactive phase plane tool (accessible from both shell and graphical interfaces): the blue line corresponds to a trajectory of a single oscillator node isolated and without noise, 4th order Runge-Kutta integration scheme**. In the bottom panel, the corresponding trajectories of both the *v*(*t*) and *w*(*t*) state variables of the model are shown. The activity exhibits oscillations at approximately 40 Hz.

**Figure 9:**
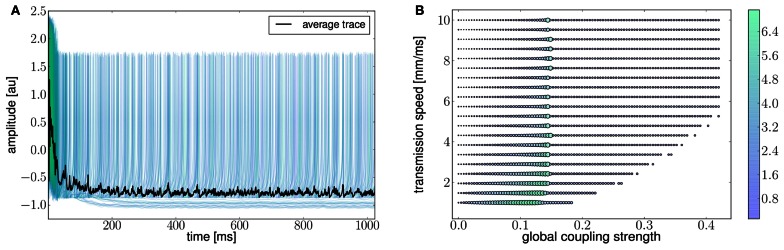
**(A)** The activity of individual regions are illustrated in colored lines. The black line represents the average activity over the network nodes. Here brain regions are weakly coupled changing both the collective and local dynamics of the network. **(B)** Using TVB scientific library as a python module we can conveniently run thousands of simulations in parallel on a cluster. Note that TVB parallelizes different tasks e.g., simulations and analyses, taking advantage of multi-core systems, however, it does not parallelize the processes themselves. Simultaneous simulations allow for a systematic parameter space exploration to rapidly gain insights of the whole brain dynamics repertoire. In this plot, the magnitude and color scale correspond to one the variance computed over all the elements of the N-dimensional output array (*Global Variance*). Simulations were performed on a cluster based on the Python demo scripts available in the release packages. On of the major strengths of *The Virtual Brain* is that G-Users are enabled to launch parameter sweeps through the UI without the need to know how to submit parallel jobs (see Figure 10).

**Figure 10:**
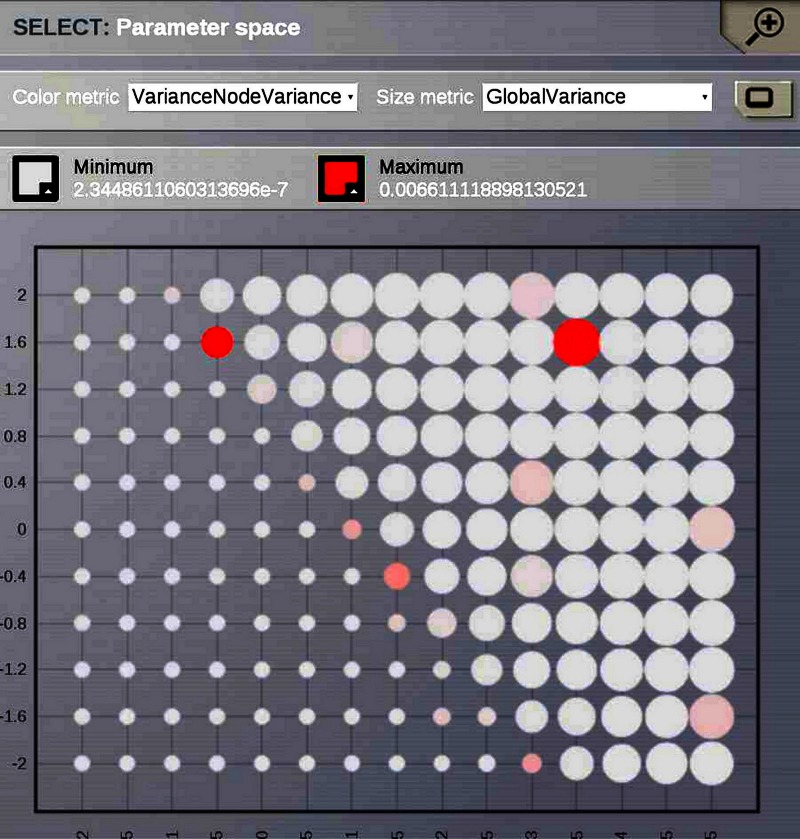
**One of TVB's major strengths is the capability to launch parallel simulations through the UI**. We show a screenshot of the resulting display when sweeping across two different parameters of the *Generic2dOscillator* model. Here each data point represents two metrics: size is mapping the *Global Variance* and color corresponds to the *Variance of the nodes Variance*. These results provide a topography of the stability space allowing users to distinguish, and thus select, combinations of critical parameters values.

Ghosh et al. (2008) and Deco et al. (2009) demonstrated the important role of three large-scale parameters in the emergence of different cluster synchronization regimes: the global coupling strength factor, time-delays (introduced via the long-range connectivity fiber tract lengths and a unique transmission speed) and noise variance. They built parameter space maps using the Kuramoto synchronization index. Here, using TVB's scripting interface, we show it is easily possible to build a similar scheme and perform a parameter space exploration in the coupling strength (*gcs*) and transmission speed (*s*) space. The ***Connectivity*** upon which the large-scale network is built was the demonstration dataset. It is bi-hemispheric and consists of 74 nodes, i.e., 37 regions per hemisphere. It includes all the cortical regions but without any sub-cortical structure such as the thalamic nuclei. Its weights are quantified by integer values in the range 0–3. The evolution of the local dynamics were represented by the model *Generic2dOscillator*, configured in such a way that a single isolated node exhibited 40 Hz oscillations (Figure 8). The variance of the output time-series was chosen as a simple, yet informative measure to represent the collective dynamics (Figure 9A) as a function of the parameters under study. Results are shown in Figure 9B. Parameter sweeps can also be launched from TVB web-interface (see Figure 10 for an illustration).

Currently TVB provides two scalar metrics based on the variance of the output time-series to perform data reduction when exploring a certain parameter space. These are *Variance of the nodes Variances* and *Global Variance*. The former zero-centers the output time-series and computes the variance over time of the concatenated time-series of each state variable and mode for each node and subsequently the variance of the nodes variances is computed. This metric describes the variability of the temporal variance of each node. In the latter all the time-series are zero-centered and the variance is computed over all data points contained in the output array.

With this example we intended to expose the possibility to reproduce workflows, i.e., modeling schemes, found in the literature. TVB is a modeling platform providing a means of cross-validating scientific work by encouraging reproducibility of the results.

### 3.3. Higher-level simulation scenarios using stimulation protocols

As one possible use case, we have set up an example based on the scheme used in McIntosh et al. (2010). The goal is to demonstrate how to build stimulation patterns in TVB, use them in a simulation, obtain EEG recordings of both the activity similar to the resting state (RS) and to evoked responses (ER), and finally make a differential analysis of the complexity of the resulting time-series by computing MSE.

In vision neuroscience, the two-stream hypothesis (Schneider, 1969) suggests the existence of two streams of information processing, the ventral and the dorsal stream. In one of these pathways, the ventral stream, the activity from subcortical regions project to V1 and the activity propagates to the temporal cortices through V2 and V4 (Goodale and Milner, 1992). We systematically stimulated the area corresponding to the primary visual cortex (V1) to demonstrate the functioning of TVB stimulation ***Patterns*** and observed how the activity elicited by a periodic rectangular pulse propagates to neighboring regions, especially V2.

**Figure 11:**
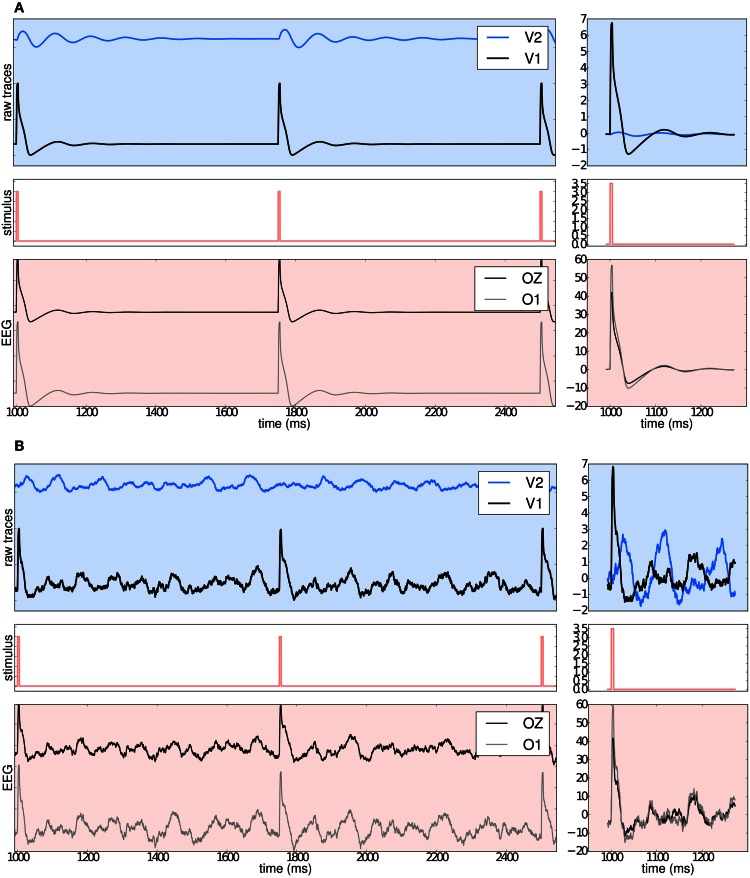
**(A)** The upper left blue panel shows the raw traces of nodes V2 and V1; the latter stimulated with a rectangular pulse of width equal to 5 ms and repetition frequency of 1 Hz. Signals are normalized by their corresponding maximum value. The right blue panel show the signals for a shorter period of time. Amplitudes are not normalized to emphasize the relative difference between the two regions. Middle panels illustrate the stimulus pattern. Lower red panels display the activity as projected onto EEG space and recorded from channels Oz and O1. The default EEG cap in TVB consists of 62 scalp electrodes distributed according to the 10–20 international system (Klem et al., 1999). In this simulation a deterministic integration scheme was employed to obtain the time-series of neural activity, since noise was not applied to the model's equations. **(B)** The same description as in **(A)** applies. The main difference with the previous simulation is that here white noise was added to the system.

Benefiting from TVB's flexibility we show in Figure 11 that it is possible to systematically stimulate a specific brain region (e.g., V1) and to highlight the anatomical connection to its target region (e.g., V2) by observing the arrival of the delayed activity; analyze the responses of the model; handle multi-modal simulated data; and extract metrics from computationally expensive algorithms to characterize both the “resting” and “evoked” states.

Currently, TVB permits the stimulation and read-out of activity from any brain area defined in the anatomical parcellation used to derive the connectome. This modeling example was built imposing a strong restriction on the number of regions to stimulate, since global dynamics can quickly become complex. Additionally, to demonstrate the many scenarios that can be set up in TVB, we simulated the same brain network model under the influence of a stimulus, first without noise (Figure 11A: using Heun deterministic method) and then with white noise (Figure 11B: using Heun stochastic method). The first approach makes it easier to see the perturbations induced by the stimulus and the propagation of activity from one region to the other. The second approach is a more realistic representation of the neural activity.

**Figure 12:**
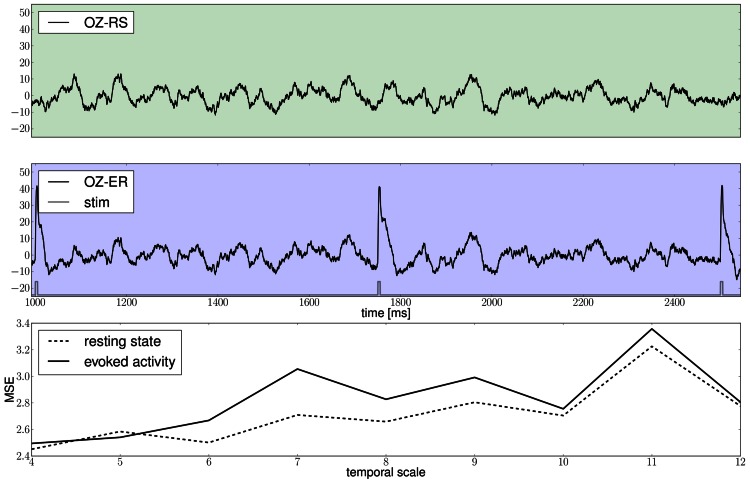
**The green and blue panels show EEG recordings from electrode Oz during the resting state, i.e., in the absence of stimulation and in the stimulated condition, respectively, notice the slow damped oscillations after stimulus onset at a approximately 10 Hz; the light gray trace depicts the stimulation pattern**. The bottom panel displays multiscale entropy estimates computed on the Oz time-series at different temporal scales using the dataset obtained by means of a stochastic integration scheme.

Results of the proposed modeling protocol are presented in Figure 12 where the EEG traces from channel Oz for the resting and evoked states are shown together with the MSE estimates.

Scripts to reproduce results from Figures 11, 12 are available in the distribution packages of TVB.

With the availability of surface-based simulations the challenge of replicating topographic maps of different sensory systems, such as those found in the primary visual cortex (Hinds et al., 2009), could be addressed.

### 3.4. Dynamic modeling

From both the shell and web interface it is possible to exploit another feature of TVB: namely, simulation continuation, i.e., a simulation can be stopped allowing users to modify model parameters, scaling factors, apply or remove stimulation or spatial constraints (e.g., local connectivity), or make any other change that does not alter the spatiotemporal domain of the system or its output (integration step, transmission speed and spatial support) and then resumed without the need of creating a new *Simulator* instance. Furthermore, this capability opens the possibility to dynamically update the simulation at runtime. Such a dynamic approach leads toward an adaptive modeling scheme where stimuli and other factors may be regulated by the ongoing activity (this last feature can be handled only from the scripting interface for the moment).

## 4. Discussion

We have presented the architecture and usage of TVB, a neuroinformatics platform developed for simulations of network models of the full brain. Its scientific core has been developed by integrating concepts from theoretical, computational, cognitive and clinical neuroscience, with the aim to integrate neuroimage modalities along with the interacting mesoscopic and macroscopic scales of a biophysical model of the brain. From a computational modeling perspective TVB constitutes an alternative to approaches such as the work of Riera et al. (2005) and more recently that of Valdes-Sosa et al. (2009), as well as other relevant studies mentioned in the main text of this article. From a neuroinformatics perspective, TVB lays the groundwork for the integration of existing paradigms in the theory of large-scale models of the brain, by providing a general and flexible framework where the advantages and limitations of each approach may be determined. It also provides the community with a technology, that until now had not been publicly available, accessible by researchers with different levels and backgrounds, enabling systematic implementation and comparison of neural mass and neural field models, incorporating biologically realistic connectivity and cortical geometry and with the potential to become a novel tool for clinical interventions. While many other environments simulate neural activity at the level of neurons (Brian simulator, MOOSE, PCSIM, NEURON, NEST, GENESIS) (Hines and Carnevale, 2001; Gewaltig and Diesmann, 2007; Goodman and Brette, 2008; Ray and Bhalla, 2008; Pecevski et al., 2009; Brette and Goodman, 2011), even mimicking a number of specific brain functions (Eliasmith et al., 2012), they, most importantly, do not consider the space-time structure of full brain connectivity constraining whole brain neurodynamics, as a crucial component in their modeling paradigm. Other approaches to multi-modal integration such as Statistical Parametric Mapping (SPM) perform statistical fitting to experimental data at the level of a small set of nodes (Friston et al., 1995, 2003; David et al., 2006; Pinotsis and Friston, 2011) [i.e., they are data-driven as in Freestone et al. (2011)], thus diverging from our approach that could be categorized as a purely “computational neural modeling” paradigm as described in Bojak et al. (2011). From this perspective, the goal is to capture and reproduce whole brain dynamics by building a network constrained by its structural large-scale connectivity and mesoscopic models governing the nodes intrinsic dynamics.

Also, the extension of neuronal level modeling to large brain structures requires vast supercomputers to emulate the large number of complex functional units. Focusing on the brain's large-scale architecture, in addition to the dimension reduction accomplished through the mean field methods applied on the mesoscopic scale, TVB allows for computer simulations on the full brain scale on workstations and small computing clusters, with no need to use supercomputing resources.

The simulator component of TVB has the goal of simulating mesoscopic neural dynamics on large-scale brain networks. It does not intend to build brain models at the level of neurons (Goodman and Brette, 2009; Cornelis et al., 2012), however, it does leverage information from microscopic models to add detail and enhance the performance of the neural population models, which act as building blocks and functional units of the network. TVB thus represents a unique tool to systematically investigate the dynamics of the brain, emphasizing its large-scale network nature and moving away from the study of isolated regional responses, thereby considering the function of each region in terms of the interplay among brain regions. The primary spatial support (neuroanatomical data) on top of which the large-scale network model is built has a number of implications:

1. It constraints the type of network dynamics; dynamics that could be further related to physiology and behavior (Senden et al., 2012).
2. It permits a systematic investigation of the consequences of the particular restrictions imposed by that large-scale structure and the effect of changes to it.
3. It provides a reliable and geometrically accurate model of sources of neural activity, enabling realistic forward solutions to EEG/MEG based on implementations of boundary element methods (BEM) or other approaches such as finite difference time domain methods (FDTD).

On the basis of the literature, theoretical and clinical studies seeking to better understand and describe certain brain functions and structure use stimulation as an essential part of their protocols. Stimulation is a way to probe how the system respond under external perturbations adapting itself to the new environmental conditions or to categorize responses when stimulation represents real-life (visual, auditory, motor) sensory inputs. Among the current features of TVB, the easy generation of a variety of stimulation patterns is to be recognized as one of its great advantages and contributions to experimental protocol design. TVB permits the development of simple stimulation routines, allowing evaluation of the viability and usefulness of certain stimulation procedures.

TVB represents a powerful research platform, combining experimental design and numerical simulations into a collaborative framework that allows sharing of results and the integration of data from other applications. Naturally, this leads to the potential for an increased level of interaction among researchers of the broad neuroscience community. In the same direction, TVB is also an extensible validation platform since it supports the creation of basic modeling refinement loops, making model exploration and validation a relatively automated procedure. For instance, after generating a brain network model, exploring the system's parameter space by adjusting parameters of both the local dynamics and the large scale structure can be achieved with ease. Further, effects of local dynamics and network structure can be disentangled by evaluating distinct local dynamic models on the same structure or the same local dynamic model coupled through distinct structures. This constrained flexibility makes it easy for modelers to test new approaches, directly compare them with existing approaches and reproduce their own and other researchers' results. Reproducibility is indeed a required feature to validate and consequently increase the reliability of scientific work (Donoho, 2010) and the extensibility of TVB's scientific components, granted by its modular design, provides a mechanism to help researchers achieve this.

The brain network models of TVB, being built on explicit anatomical structure, enable modeling investigations of practical clinical interest. Specifically, whenever a dysfunction or disease expresses itself as a change to the large scale network structure, for instance, in the case of lesions in white-matter pathways, the direct replication of this structural change in TVB's brain network models is straight forward.

## Future work

Regarding performance, of special importance will be to evaluate all the parameters that have an effect on both memory usage and execution time for surface-based simulations. The reason is that realistic brain network models are built on top of surface meshes constructed by thousands of vertices per hemisphere (2^13^ for the TVB demonstration cortical surface) but can easily have more than 40,000.

Equally important is to develop more tests to generally evaluate the simulation engine, paying close attention to keep the consistency and stability of the algorithms currently implemented.

Another aspect that deserves careful attention is the description of our modeling approach that was largely beyond the scope of this text. Therefore, the theory underlying the different methods involved in the development of a generalized framework for brain network models is to be presented in future scientific publications.

To allow a most optimal dissemination of knowledge in TVB we are currently developing a web-based educational platform that will allow training on the usage of TVB, as well as serve as a key reference.

As simulations in TVB are built on the large-scale anatomical structure of the human brain, continued work to integrate new, reliable, sources of structural data is essential to the progress of the platform. An obvious future resource in this regard will be the newly developed database of the Human Connectome Project (Essen and Ugurbil, 2012; Essen et al., 2012).

## Information sharing statement (license)

The data and software in this study belong to an ongoing project; it is free software and licensed under the GNU General Public License version 2 as published by the Free Software Foundation. The latest releases of *The Virtual Brain* including the source code and demo data are free to download from http://www.thevirtualbrain.org. The source code available in the public repository includes the latest experimental features regarding GPU implementation.

### Conflict of interest statement

The authors declare that the research was conducted in the absence of any commercial or financial relationships that could be construed as a potential conflict of interest.
