# Supplementary material for: The Virtual Brain: a simulator of primate brain network dynamics
Source: Front Neuroinform. 2013 Jun 11;7:10. doi: 10.3389/fninf.2013.00010 (PMC3678125; doi:10.3389/fninf.2013.00010)
Supplement: Supplementary file 1 [file DataSheet1.PDF]

Supplementary Code 1: The *Generic2dOscillator* model as a template to include a new model in TVB.

```
# -*- coding: utf-8 -*-
"""
A template for integrating a new model  using the default
Generic2dOscillator with complete docstrings and comments.

.. moduleauthor:: TVB-Team

"""

# Third party python libraries
import numpy
import numexpr

#The Virtual Brain
from tvb.simulator.lab import *
import tvb.datatypes.arrays as arrays
import tvb.basic.traits.types_basic as basic
import tvb.simulator.models as models


class Generic2dOscillator(models.Model):
    """
    The Generic2dOscillator model is a generic dynamic system with two
    state variables. The dynamic equations of this model are composed
    of two ordinary differential equations comprising two nullclines.
    The first nullcline is a cubic function as it is found in most
    neuron and population models; the  second nullcline is arbitrarily
    configurable as a polynomial function up to second order. The
    manipulation of the latter nullcline's parameters allows to
    generate a wide range of different behaviors.
    See:

        .. [FH_1961] FitzHugh, R., *Impulses and physiological states in
            theoretical models of nerve membrane*, Biophysical Journal 1: 445, 1961.

        .. [Nagumo_1962] Nagumo et.al, *An Active Pulse Transmission Line
            Simulating Nerve Axon*, Proceedings of the IRE 50: 2061, 1962.

        .. [SJ_2011] Stefanescu, R., Jirsa, V.K. *Reduced representations of
            heterogeneous mixed neural networks with synaptic coupling*.
            Physical Review E, 83, 2011.

        .. [SJ_2010]    Jirsa VK, Stefanescu R.  *Neural population modes
            capture biologically realistic large-scale network dynamics*.
            Bulletin of Mathematical Biology, 2010.

        .. [SJ_2008_a] Stefanescu, R., Jirsa, V.K. *A low dimensional
            description of globally coupled heterogeneous neural
            networks of excitatory and inhibitory neurons*. PLoS
            Computational Biology, 4(11), 2008).

    The model's (:math:`V`, :math:`W`) time series and phase-plane
    its nullclines can be seen in the figure below. The model with
    its default parameters exhibits FitzHugh-Nagumo like dynamics.

     ---------------------------
    |  EXCITABLE CONFIGURATION  |
     ---------------------------
    |Parameter     |  Value     |
    -----------------------------
    | a            |     -2.0   |
    | b            |    -10.0   |
    | c            |      0.0   |
    | d            |      0.1   |
    | I            |      0.0   |
    -----------------------------
    |* limit cylce if a = 2.0   |
    -----------------------------

     ---------------------------
    |   BISTABLE CONFIGURATION  |
     ---------------------------
    |Parameter     |  Value     |
    -----------------------------
    | a            |      1.0   |
    | b            |      0.0   |
    | c            |     -5.0   |
    | d            |      0.1   |
    | I            |      0.0   |
    -----------------------------
    |* monostable regime:       |
    |* fixed point if Iext=-2.0 |
    |* limit cycle if Iext=-1.0 |
    -----------------------------
```

```
     ---------------------------
    |  EXCITABLE CONFIGURATION  | (similar to Morris-Lecar)
     ---------------------------
    |Parameter     |  Value     |
    -----------------------------
    | a            |      0.5   |
    | b            |      0.6   |
    | c            |     -4.0   |
    | d            |      0.1   |
    | I            |      0.0   |
    -----------------------------
    |* excitable regime if b=0.6|
    |* oscillatory if b=0.4     |
    -----------------------------


     ---------------------------
    |  SanzLeonetAl  2013       |
     ---------------------------
    |Parameter     |  Value     |
    -----------------------------
    | a            |    - 0.5   |
    | b            |    -15.0   |
    | c            |      0.0   |
    | d            |      0.02  |
    | I            |      0.0   |
    -----------------------------
    |* excitable regime if      |
    |* intrinsic frequency is   |
    |  approx 10 Hz             |
    -----------------------------

    """


    _ui_name = "Generic 2d Oscillator"
    ui_configurable_parameters = ['tau', 'a', 'b', 'c', 'd', 'I']

    #Define traited attributes for this model, these represent possible kwargs.
    tau = arrays.FloatArray(
        label = r":math:`\tau`",
        default = numpy.array([1.0]),
        range = basic.Range(lo = 0.00001, hi = 5.0, step = 0.01),
        doc = """A time-scale hierarchy can be introduced for the state
        variables :math:`V` and :math:`W`. Default parameter is 1, which means
        no time-scale hierarchy.""",
        order = 1)

    I = arrays.FloatArray(
        label = ":math:`I_{ext}`",
        default = numpy.array([0.0]),
        range = basic.Range(lo = -2.0, hi = 2.0, step = 0.01),
        doc = """Baseline shift of the cubic nullcline""",
        order = 2)

    a = arrays.FloatArray(
        label = ":math:`a`",
        default = numpy.array([-2.0]),
        range = basic.Range(lo = -5.0, hi = 5.0, step = 0.01),
        doc = """Vertical shift of the configurable nullcline""",
        order = 3)

    b = arrays.FloatArray(
        label = ":math:`b`",
        default = numpy.array([-10.0]),
        range = basic.Range(lo = -20.0, hi = 15.0, step = 0.01),
        doc = """Linear slope of the configurable nullcline""",
        order = 4)

    c = arrays.FloatArray(
        label = ":math:`c`",
        default = numpy.array([0.0]),
        range = basic.Range(lo = -10.0, hi = 10.0, step = 0.01),
        doc = """Parabolic term of the configurable nullcline""",
        order = 5)

    d = arrays.FloatArray(
        label = ":math:`d`",
        default = numpy.array([0.1]),
        range = basic.Range(lo = 0.0001, hi = 1.0, step = 0.0001),
        doc = """Temporal scale factor.""",
        order = -1)

    e = arrays.FloatArray(
        label = ":math:`e`",
        default = numpy.array([3.0]),
        range = basic.Range(lo = -5.0, hi = 5.0, step = 0.0001),
        doc = """Coefficient of the quadratic term of the cubic nullcline.""",
        order = -1)

    f = arrays.FloatArray(
```

```
        label = ":math:`f`",
        default = numpy.array([1.0]),
        range = basic.Range(lo = -5.0, hi = 5.0, step = 0.0001),
        doc = """Coefficient of the cubic term of the cubic nullcline.""",
        order = -1)

    alpha = arrays.FloatArray(
        label = ":math:`\alpha`",
        default = numpy.array([1.0]),
        range = basic.Range(lo = -5.0, hi = 5.0, step = 0.0001),
        doc = """Constant parameter to scale the rate of feedback from the
            slow variable to the fast variable.""",
        order = -1)

    beta = arrays.FloatArray(
        label = ":math:`\beta`",
        default = numpy.array([1.0]),
        range = basic.Range(lo = -5.0, hi = 5.0, step = 0.0001),
        doc = """Constant parameter to scale the rate of feedback from the
            slow variable to itself""",
        order = -1)

    #Informational attribute, used for phase-plane and initial()
    state_variable_range = basic.Dict(
        label = "State Variable ranges [lo, hi]",
        default = {"V": numpy.array([-2.0, 4.0]),
                   "W": numpy.array([-6.0, 6.0])},
        doc = """The values for each state-variable should be set to encompass
            the expected dynamic range of that state-variable for the current
            parameters, it is used as a mechanism for bounding random initial
            conditions when the simulation isn't started from an explicit
            history, it is also provides the default range of phase-plane plots.""",
        order = 6)


    variables_of_interest = basic.Enumerate(
        label = "Variables watched by Monitors",
        options = ["V", "W"],
        default = ["V",],
        select_multiple = True,
        doc = """This represents the default state-variables of this
            Model to be monitored. It can be overridden for each
            Monitor if desired. The corresponding state-variable
            indices for this model are :math:`V = 0`and :math:`W = 1`.""",
        order = 7)


    def __init__(self, **kwargs):
        """
        Intialise Model
        """

        LOG.info("%s: initing..." % str(self))

        super(Generic2dOscillator, self).__init__(**kwargs)

        self._nvar = 2
        # long range coupling variables
        self.cvar = numpy.array([0], dtype=numpy.int32)

        LOG.debug("%s: inited." % repr(self))


    def dfun(self, state_variables, coupling, local_coupling=0.0,
             ev=numexpr.evaluate):
        r"""
        The two state variables :math:`V` and :math:`W` are typically considered
        to represent a function of the neuron's membrane potential, such as the
        firing rate or dendritic currents, and a recovery variable, respectively.
        If there is a time scale hierarchy, then typically :math:`V` is faster
        than :math:`W` corresponding to a value of :math:`\tau` greater than 1.

        The equations of the generic 2D population model read

        .. math::
            \dot{V} &= \tau (\alpha W - V^3 +3 V^2 + I) \\
            \dot{W} &= (a\, + b\, V + c\, V^2 - \, beta W) / \tau

        where external currents :math:`I` provide the entry point for local,
        long-range connectivity and stimulation.

        """

        V = state_variables[0, :]
        W = state_variables[1, :]

        #[State_variables, nodes]
        c_0 = coupling[0, :]

        tau = self.tau
        I = self.I
```

```
        a = self.a
        b = self.b
        c = self.c
        d = self.d
        e = self.e
        f = self.f
        beta  = self.beta
        alpha = self.alpha

        lc_0 = local_coupling*V

        ## numexpr
        dV = ev('d * tau * (alpha * W - f * V**3 + e * V**2 + I + c_0 + lc_0)')
        dW = ev('d * (a + b * V + c * V**2 - beta * W) / tau')

        self.derivative = numpy.array([dV, dW])

        return self.derivative


if __name__ == "__main__":

    #Initialise Model in their default state:
    G2D_MODEL = Generic2dOscillator()

    LOG.info("Model initialised in its default state without error...")
    LOG.info("Testing phase plane interactive ... ")

    # Check local dynamics
    from tvb.simulator.plot.phase_plane_interactive import PhasePlaneInteractive
    import tvb.simulator.integrators as integrators

    INTEGRATOR = integrators.HeunDeterministic(dt=2**-4)
    ppi_fig = PhasePlaneInteractive(model=G2D_MODEL, integrator=INTEGRATOR)
    ppi_fig.show()
```

Notice that this template includes all the documentation related to the particular model that is defined, following the docstrings conventions (http://www.python.org/dev/peps/pep-0257/). The model state variables are defined in the *dfun* method.
